# Moment-based metrics for molecules computable from cryogenic electron microscopy images

**DOI:** 10.1017/S2633903X24000023

**Published:** 2024-02-23

**Authors:** Andy Zhang, Oscar Mickelin, Joe Kileel, Eric J. Verbeke, Nicholas F. Marshall, Marc Aurèle Gilles, Amit Singer

**Affiliations:** 1 Program in Applied and Computational Mathematics, Princeton University, Princeton, NJ, USA; 2 Department of Mathematics and Oden Institute, University of Texas at Austin, Austin, TX, USA; 3 Department of Mathematics, Oregon State University, Corvallis, OR, USA; 4 Department of Mathematics, Princeton University, Princeton, NJ, USA

**Keywords:** alignment-free metric, Kam’s method, method of moments, protein structural similarity, rotationally invariant distance, structural search

## Abstract

Single-particle cryogenic electron microscopy (cryo-EM) is an imaging technique capable of recovering the high-resolution three-dimensional (3D) structure of biological macromolecules from many noisy and randomly oriented projection images. One notable approach to 3D reconstruction, known as Kam’s method, relies on the moments of the two-dimensional (2D) images. Inspired by Kam’s method, we introduce a rotationally invariant metric between two molecular structures, which does not require 3D alignment. Further, we introduce a metric between a stack of projection images and a molecular structure, which is invariant to rotations and reflections and does not require performing 3D reconstruction. Additionally, the latter metric does not assume a uniform distribution of viewing angles. We demonstrate the uses of the new metrics on synthetic and experimental datasets, highlighting their ability to measure structural similarity.

## Impact Statement

Single-particle cryogenic electron microscopy (cryo-EM) is a popular method to obtain three-dimensional (3D) reconstructions of biological molecules from noisy two-dimensional (2D) tomographic projection images. Many iterative techniques for this reconstruction require initializations sufficiently close to the unknown structure to obtain high-quality reconstructions. To help select an initialization from a database of known structures, this paper introduces a metric to compare the similarity of known 3D structures with a stack of noisy 2D tomographic projection images of an unknown structure. We show that this metric distinguishes differing structures and present an efficient method to compute it, notably without performing 3D reconstruction.

## 1. Introduction

Single-particle cryogenic electron microscopy (cryo-EM) enables high-resolution reconstruction of three-dimensional (3D) biological macromolecules from a large collection of noisy and randomly oriented projection images. Kam’s method^(^^1^^)^ is one of the earliest methods proposed for homogeneous reconstruction in cryo-EM. It is a statistical method-of-moments approach applied to the cryo-EM reconstruction problem, where the computation of low-order statistics of two-dimensional (2D) images allows for the estimation of 3D structure through solving a polynomial system. Kam’s method has helped push the theoretical understanding of the reconstruction process – under certain conditions, it is a provable algorithm and provides bounds for the estimated structure’s quality in terms of the noise level and the number of images.^(^^2^ ^–^^8^ ^)^ Kam’s method also enjoys other remarkable properties:

1. It bypasses the need for angular assignment, typically a large computational burden in competing methods.
2. It is a streaming algorithm and is thus theoretically much faster than iterative methods.
3. It can – in theory – break the detection limit of the minimal size of proteins that can be observed in cryo-EM.^(^^9^ ^)^

While theoretically attractive, Kam’s method has not been able to yield high-resolution reconstructions as yet. One direction that is currently being explored to resolve this issue is the development of better priors, for instance, based on the sparsity of the solution as in the study by Bendory et al.^(^^7^ ^)^ Separately, there has been considerable, continued interest in utilizing the vast amount of solved structures stored in the Protein Data Bank (PDB)^(^^10^ ^)^ to improve cryo-EM reconstructions.

Leveraging the PDB as a prior, we propose a method to match either projection images or molecular volumes to a database of previously solved structures (Section 3). We use this procedure as a rotationally and reflectionally invariant metric that can be directly computed from raw image datasets without needing a 3D reconstruction process. Importantly, our metric neither relies on prior knowledge of rotations nor assumes a uniform rotational distribution, making it applicable to typical datasets.

To demonstrate the efficacy of our metric, we compare it to existing methods and show empirically that it achieves similar performance to alignment-based metrics. As an application, we use our metric to compute a low-dimensional embedding of a subset of the PDB into the Euclidean plane, visually showcasing how structurally similar proteins are embedded near each other (Section 4.2). Further, we apply the version of the metric that can be directly computed from stacks of 2D images and show that it gives an efficient methodology to identify the nearest neighbors in a database to a given set of experimental moments on synthetic and real datasets (Sections 4.3 and 4.4).

## 2. Background

This section presents the mathematical preliminaries needed to define our metric. Let 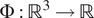 be the electrostatic potential of a molecule and 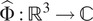 be its Fourier transform, which we define by

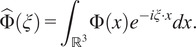

A single projection image is given by

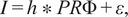

and its Fourier transform is

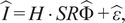

where 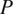 is the projection operator, 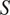 is the slicing operator, 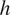 is a point spread function, 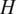 is the contrast transfer function (CTF), 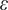 is noise, and 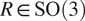 is a rotation operator. We assume that the Fourier transform 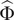 can be expanded in a spherical harmonic expansion:
(1)

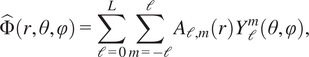

where 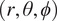 are spherical coordinates, and 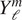 denotes the complex spherical harmonic:

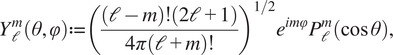

where 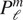 are the associated Legendre polynomials, 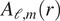 are 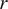 -dependent coefficients, and 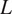 is a bandlimit parameter. See Eq. 14.30.1 in^(^^11^ ^)^ for the definitions of 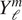 and 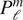 .

Let 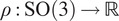 be the probability density function of the rotational distribution, which without loss of generality is invariant to in-plane rotations and reflections. (Note that by augmenting the dataset with in-plane rotations and reflections of all 2D images, one can always reduce to the case of such an invariant distribution 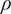 ; for example, see the study by Ponce and Singer^(^^12^ ^)^). More formally, 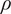 is a function on 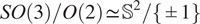 _,_ which is formed by identifying antipodal points on the sphere 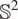 _._^(^^13^ ^)^ Thus, we model the density as a function 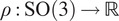 with an even-degree spherical harmonic expansion:
(2)

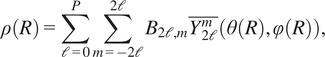

where 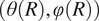 represents the third column of the rotation matrix given by 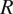 in spherical coordinates, and 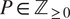 is a bandlimit parameter (see Section 4.2 in the study by Sharon et al.^(^^8^ ^)^). The analytical first and second moments 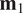 and 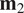 of the Fourier-transformed projection images with respect to 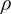 are
(3)

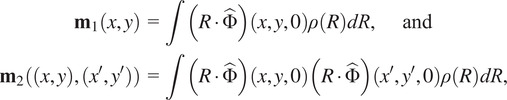

where 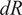 denotes integration with respect to the Haar measure on 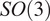 . It will be convenient to write 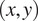 and 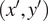 in terms of polar coordinates 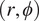 and 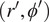 , respectively. In Appendix A.1, we show in (12) and (13) that the first moment only depends on 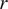 , that is, 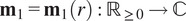 , and that the second moment only depends on 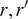 and 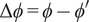 , that is, 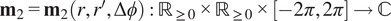 . We write 
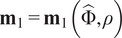
 and 
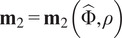
 to denote the first and second moments defined by 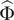 and 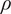 when discussing multiple structures. The basis of Kam’s method is that the moments in (3) can be estimated from images and related to expansion coefficients for the potential 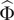 ; see Appendix A for explicit formulas.

## 3. Definition of Kam’s metrics

We now use metrics between the moments in (3) to define similarity between proteins and stacks of images. A first function is used to measure the similarity of two known structures by the moments of their potential as defined in (3). The second is used to measure the similarity between a known structure and the unknown structure observed in a dataset of images.

Crucially, the metrics can be computed without performing 3D alignment of the structures, reducing their computational costs compared to other approaches. Moreover, one of the metrics can be directly computed from noisy and CTF-affected projection images. This enables a nearest neighbor search among known structures to determine an initialization for the 3D reconstruction pipeline, especially in the expectation–maximization procedure.^(^^14^ ^,^^15^ ^)^

### Kam’s volume metric 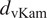


3.1.

Here, we introduce the first of Kam’s metrics, which measures the similarity of two 3D structures. We use this to perform dimensionality reduction to visualize the relationship between structures from a subset of the PDB.

In detail, given two 3D structures 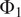 and 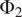 , we define the distance between them through their first and second moments 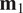 and 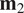 under a uniform distribution of viewing directions, which we denote by 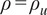 . We will derive the explicit equations for the uniform case in (17) and (18). We then measure the resulting weighted deviation of the first and second moments by
(4)

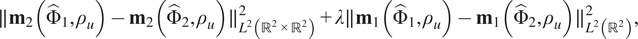

 where 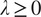 is a hyperparameter that we set to 1 for all experiments. The moments will be represented on a discretized voxel grid, and we therefore replace the continuous norms with discrete norms. More specifically, we will represent the second moment using a grid 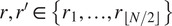 and 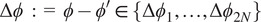 , where 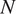 is the number of pixels of one side of the discretized volume. We define the grid points 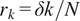 , 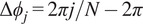 , for 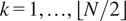 , and 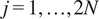 , where 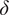 is the side length of the volume grid in angstroms. We then use the following two approximations to the continuous norms above
(5)

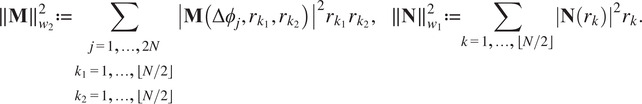

With these norms, we define the metric comparing two sets of moments of two 3D structures by
(6)

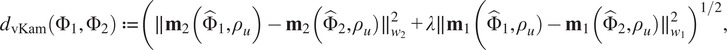



This distance is rotationally invariant since for any rotation 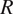 , we have 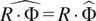 and the moments 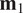 and 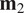 in (2) satisfy
(7)

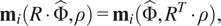

as can be seen through a change of variables in (3). When 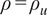 is uniform, clearly 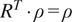 , which therefore shows rotational invariance of the cost function in (4), up to the discretization of the volume grid. Note that this bypasses the need for an alignment step. We detail the procedure for computing 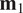 , 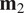 and therefore 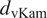 in Appendix A.1. Under certain conditions, it has been demonstrated that the second moment of the image collection identifies the 3D structure uniquely^(^^2^ ^–^^4^ ^,^^6^ ^,^^7^ ^)^ or up to a finite list of candidate structures.^(^^8^ ^)^ In Section 4.2, we show that our metric is alike other similarity scores but remarkably does not rely on alignment.

### Kam’s image metric 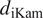


3.2.

We now introduce a metric between the empirical moments computed from a set of experimental projection images and the moments computed from the atomic coordinates of a known structure that compares images to the known structure. We detail the procedure for computing these moments in Appendix A.1.

If the distribution of poses in the experimental dataset would be known to be uniform, the empirical moments could directly be substituted for 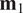 and 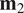 in (6) and the metric could be defined as the deviation between the moments of the two structures. In practice, however, the distribution of angles is not uniform and is unknown. Since the moments are functions of this distribution, it must therefore be inferred.

We will show in (12) and (13) that 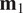 and 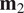 depend linearly on the expansion coefficients 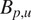 of the distribution of viewing directions. The optimization problem minimizing the discrepancy between the moments of the two structures is, therefore, a linear least-squares problem in 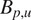 . It follows from Table 3 of^(^^8^ ^)^ that this linear least-squares is Zariski-generically full-rank (although not necessarily well-conditioned) for various small bandlimits 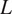 and 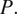 Solving this optimization problem efficiently eliminates the unknown rotational distribution. We then define the metric between the moments of the structure 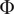 and the experimental moments 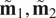 by
(8)

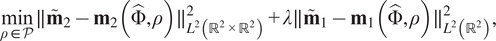

where 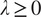 is a hyperparameter which we set to 1 for all experiments and
(9)

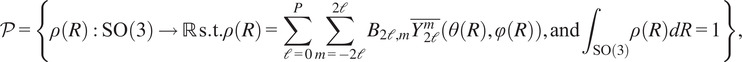

is the set of admissible distributions of viewing directions that are invariant to global reflections and in-plane rotations, where 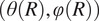 is as in (2). To simplify the optimization problem and lead to faster algorithms, note that we do not impose positivity of the distributions 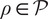 , though this could be enforced, for instance, by imposing linear constraints 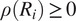 for a suitable choice of 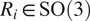 . Moreover, the constraint 
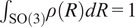
 is equivalent to imposing 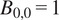 _,_^(^^8^ ^)^ which can be achieved by removing 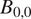 from the set of optimization variables and fixing its value to 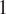 . The values of the bandlimit parameters 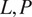 and the hyperparameter 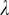 used in our numerical experiments are given in Appendix B.1.

Just as in the previous section, we replace the continuous norms in (8) by discrete norms to define the metric between empirical moments and the moments from a 3D structure as
(10)

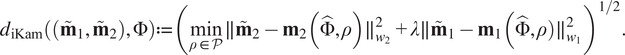



The cost function in (8) is rotationally invariant, in that it does not depend on the orientation of 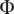 , since (7) implies that
(11)

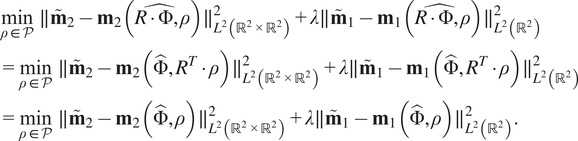

where the last equality follows because 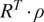 lies in 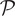 , since rotating a viewing angle distribution over 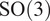 results in another viewing angle distribution over 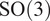 .

At the cost of solving the small linear system detailed in Appendix A.3, our method allows for the comparison between a stack of images and a resolved structure, without performing a 3D reconstruction. Furthermore, we precompute the least-squares matrices necessary for optimization, after which the distance function can be calculated in real time. With sufficient storage and precomputation, the procedure is scalable to the entirety of the PDB.

In particular, 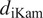 can be used in an efficient scheme to match a stack of synthetic images to the potentials of nearby PDB structures. By selecting a subset of the PDB database, one can efficiently compute 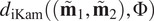 for each 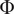 in the subset and find the nearest neighbors. The method for processing image moments in practice is detailed in Appendix A.4, and the computational complexity of the metric is derived in Appendix A.5.

## 4. Results

### 4.1. Existing measures of structural similarity

There are several existing methods for reporting structural similarity between two known volumes. We list two approaches based on computing alignment and Zernike moments. We compare both 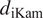 and 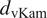 to these approaches in the experiments in the following subsections. Note that the following existing metrics are limited to measuring similarity between two structures and cannot compare images to structures, whereas 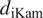 can.

1. *Euclidean alignment*: A classical approach for comparing the similarity of two structures is to sample the volumes on a 3D grid and calculate the Euclidean distance between pairs over rotations and translations. However, this method is expensive to compute since optimization over 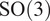 is required to align the structures. Accelerated methods for computing these alignments by maximizing the correlation between two volume maps over rotations and translations have been implemented in various programs, for example, via gradient ascent in Chimera.^(^^16^ ^)^ Further acceleration can be achieved by calculating volumetric correlations by expanding the volumes in a well-chosen basis and applying dimensionality reduction^(^^17^ ^)^ or by maximizing the correlation between common lines in projection images generated from the volumes.^(^^18^ ^)^ Similar alignment methods, such as those described in the study by Bartesaghi et al. and Xu et al.^(^^19^ ^,^^20^ ^)^, are also used in electron tomography for sub-volume similarity. In this paper, we use a Bayesian optimization algorithm to minimize an Euclidean loss function, as described in the study by Singer and Yang,^(^^21^ ^)^ to compute the alignment and minimum distance between two volumes.
2. *Zernike moments*: Another metric for structural similarity is to expand the molecule’s structure in Zernike polynomials and compute a metric from the Zernike expansion coefficients, as described in the study by Guzenko et al.^(^^22^ ^)^, which is used by the PDB for structural similarity search.

### Applying 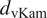 to a PDB subset

4.2.

To test the ability of 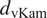 to discern the similarity between 3D structures, we first generate a database using 1420 structures downloaded from the PDB.^(^^10^ ^)^ The subset chosen here was selected by filtering for human proteins with an experimental structure at resolution between 1 and 3 Å and a molecular weight between 150 and 250 kDa. We use this subset because it encompasses a diverse range of shapes and symmetries as well as many homologous structures. Additionally, the weight range reflects a smaller and more challenging protein size for a typical cryo-EM experiment.^(^^23^ ^)^ In the future, a larger database containing the entire PDB can also be generated.

Using our database, we first generate a discretized potential for each structure as described in Appendix A.2. The first and second moments of each structure can then be computed using (3). We then compute 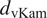 in (6) pairwise for all structures in the database.

To compare the performance of 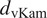 against existing metrics, we calculate pairwise scores using 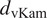 , Euclidean alignment, and the Zernike metric. We then plot the returned scores against each other and calculate a ranking similarity using normalized discounted cumulative gain^(^^24^ ^)^ (NDCG). We use this metric since it is a popular method to quantify the similarity between sets of rankings; its calculation is given in Appendix A.6.

**Figure 1.**
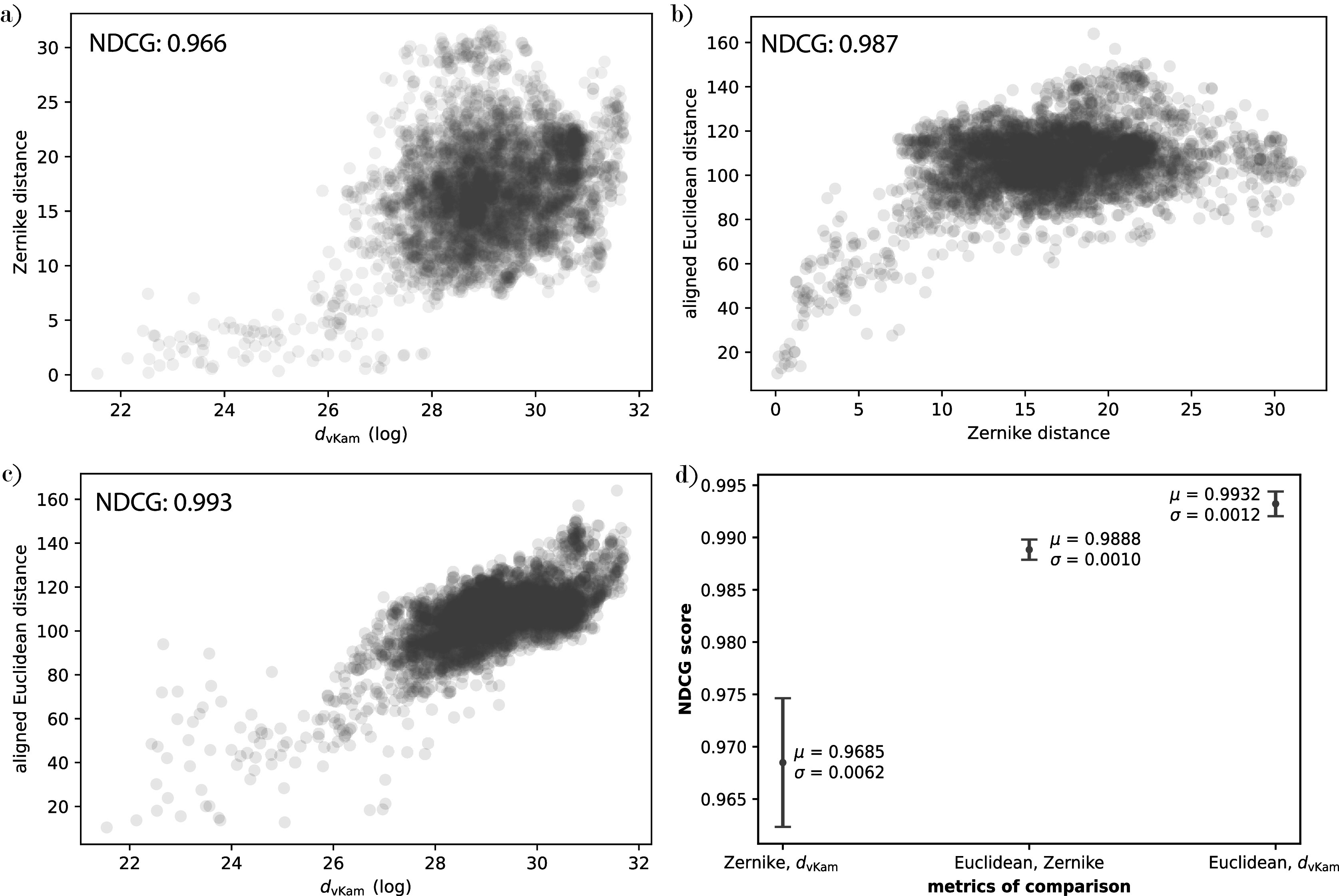
Comparison between 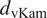 , the Zernike metric, and Euclidean alignment. a-c) A random size 100 subset of the database is selected. Then, pairwise similarity metrics are calculated and plotted, where each point represents a pair of structures. The NDCG score is calculated using the metric on the 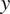 -axis as the predicted metric, and the metric on the 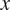 -axis as the true metric. d) The procedure is repeated with 10 randomly selected size 100 subsets, and the mean ( 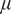 ) and standard deviation ( 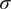 ) of the NDCG scores are calculated. The error bars and points visualize 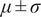 .

In Figure 1, we report the NDCG scores between pairs of metrics. All NDCG scores are close to 1, indicating strong agreement among the three different metrics on which structures are most similar. However, the alignment metric and 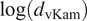 share the highest average NDCG score. To verify the statistical significance of this agreement, we report a t-test by selecting 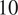 different subsets, showing that the NDCG score between 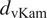 and the alignment metric is statistically significantly higher (with a *p*-value 
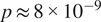
 ) than the NDCG score between the alignment metric and the Zernike metric. We thus conclude that 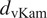 provides a fast and accurate alternative for the alignment metric.

Although it is the most interpretable metric, Euclidean alignment is computationally expensive to execute for all pairs of structures in a database. To achieve a manageable runtime for alignment, we calculate pairwise Euclidean alignment distances for a subset of the database of size 100. Pairwise alignment on this subset took 8 hours on a 2.6 GHz Intel Skylake Central Processing Unit (CPU) running AVX-512 using 16 physical cores and 80 GB random-access memory (RAM). To do pairwise alignment via Bayesian optimization for the entire database of 1420 structures would require 46 days of computation, whereas using 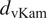 (including precomputation) to calculate pairwise distances between all 1420 structures in the database requires 3 minutes on the same hardware. Despite containing an alignment component, the Zernike metric is also fast, taking 3 minutes to compute pairwise distances for the entire database.

**Figure 2.**
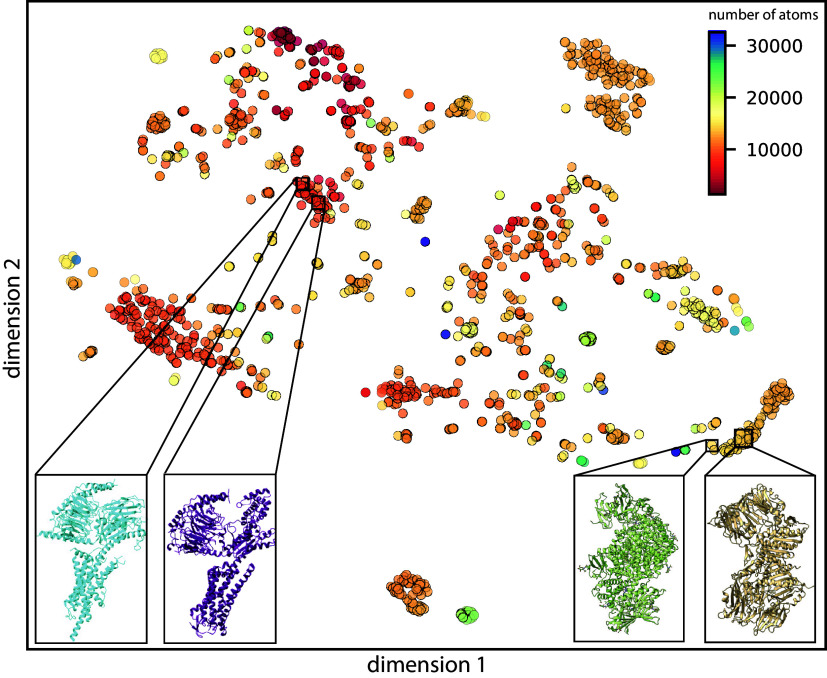
2D embedding of protein structures based on their similarity using 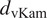 . The analytical moments of 1420 proteins were computed and compared using (6), and t-SNE was applied for visualization. Each node represents a single structure and is colored by the number of atoms. Distinct clusters containing homologous or similarly shaped structures suggest that 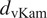 provides interpretable results.

After observing high agreement between 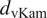 and the other metrics, we compute a 2D embedding of the similarity between structures in our database using t-distributed stochastic neighbor embedding (t-SNE)^(^^25^ ^)^ (see Figure 2). Analogous t-SNE plots for the alignment metric and Zernike metric are reported in Appendix B.2. We find that 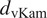 provides interpretable results in identifying similar molecules from their moments without the need for alignment. In particular, we observe that both homologous (i.e., structures with similar sequences) and similar-shaped structures are shown to be clustered together.

### Database search using 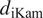 with synthetic cryo-EM data

4.3.

We next demonstrate the ability of 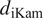 to accurately find a match for the moments computed from projection images to a database of analytical moments computed from the atomic coordinates of known structures. To test our metric, we use the same dataset as the previous section, selecting the protein structure of a Mas-related G-protein-coupled receptor (available as entry PDB-7VV3^(^^26^ ^)^) from our database described in Section 4.2. We use this entry because our database includes several similarly shaped yet nonidentical structures, on which we examine our metric’s performance.

**Figure 3.**
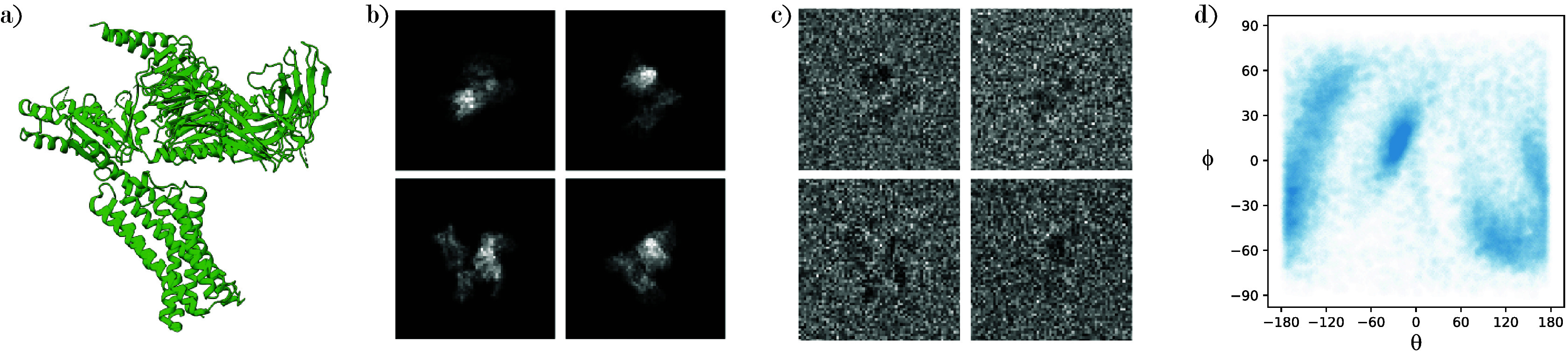
Visualization of the generation of simulated images. (a) Protein structure of PDB-7VV3. (b) Clean projection images from PDB-7VV3 generated with a nonuniform viewing angle distribution. (c) Projection images corrupted with a CTF and white noise with 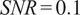 . (d) Distribution of nonuniform viewing angles.

We generate a synthetic cryo-EM dataset as illustrated in Figure 3: We take 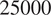 clean projection images from a nonuniform distribution over 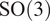 at viewing angles given by a mixture of three von Mises–Fisher distributions.^(^^27^ ^)^ To simulate cryo-EM data, the images are then corrupted with one of 100 unique radial CTFs, after which we add white noise with a signal-to-noise ratio (SNR) of 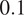 . We define the SNR by taking the signal as the average squared intensity over each pixel in all the clean images and setting the noise variance to the appropriate ratio of the signal. These simulated images are generated using the ASPIRE software package^(^^28^ ^)^ and have parameters consistent with many experimental datasets.

**Figure 4.**
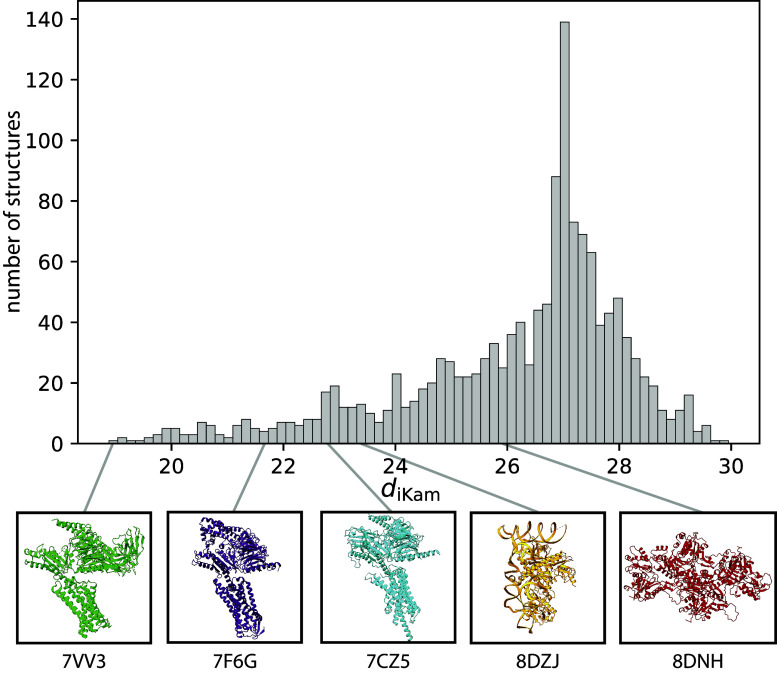
Histogram ranking of dissimilarities computed using 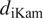 on simulated noisy projection images generated from PDB-7VV3.

We then compute the moments of the simulated images as will be shown in (12) and (13) and compare them to the database of moments using the image-to-volume metric described in (8). We also report the effect of varying the number of images on the metric’s performance in Appendix B.3. Using our metric, we can rank the similarity of the image’s moments to our database as shown in Figure 4. We show that the most similar score (*i.e.*, the smallest value in image Kam’s metric) corresponds to the ground truth structure used to generate the images. Furthermore, based on our results, the next top 116 structures correspond to structures with similar volumes and sequences. These results demonstrate that we are able to compare directly between noisy, CTF-corrupted images and known structures. This approach could be especially valuable if there is no known model for initialization in 3D reconstruction or if the molecule generating the images is unknown.^(^^29^ ^)^

**Figure 5.**
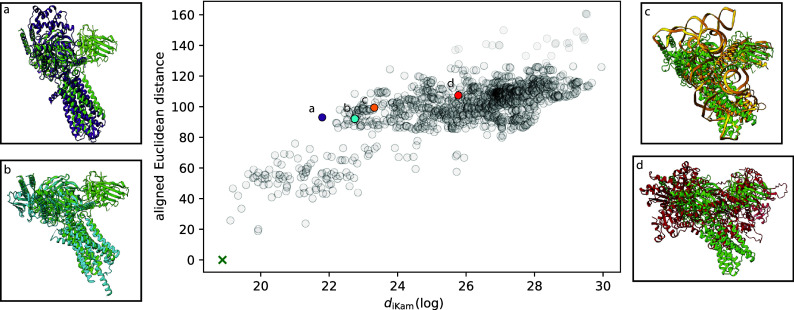
Comparison between the rankings given by 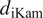 (computed from simulated images) and the minimum Euclidean distance after alignment (computed from volumes). The structures shown are superimposed with the ground truth after alignment in panels (a)–(d). The points on the graph that correspond to these structures are colored and labeled. The ground truth corresponds to the green cross in the lower left. Note that the Euclidean alignment metric shows stagnation whereas Kam’s metric does not.

We report alignment scores between molecules in our database to PDB entry 7VV3, compare these to our metric’s scores, and plot the results in Figure 5. Most notably, when the protein structure becomes less similar to the ground truth (7VV3), the alignment metric begins to lose discriminative power. Figure 5 shows structures with varying degrees of dissimilarity as having the same score ( 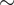 100). In contrast, our metric retains discriminative power, ranking structures with similar sequences/functions before structures with similar shapes.

Alignment via Bayesian optimization between one structure and the 1420 structures in the database took 95 minutes using the hardware described in Section 4.2. Aside from the computational cost, the interpretation of the optimal rotation returned by alignment becomes unclear when comparing two structures that are not volumetrically similar. On the other hand, our metric does not return an alignment between two structures, which could render it less useful when an explicit alignment must be computed. Without this alignment, it may become harder to visually compare their volumes.

It is computationally costly to generate and perform moment estimation on synthetic images for every molecule in the database. As such, to compare the performance of our metric against the Zernike metric, we select from our database a random subset of 100 structures. For each structure, we repeat the process we perform on PDB-7VV3: First, we generate a nonuniform distribution over 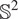 as a mixture of three von Mises–Fisher distributions with random means, weights, and covariance matrices. We then generate 25000 images, corrupt with SNR = 0.1 and radial CTFs, compute the moments, and search across the database.

**Figure 6.**
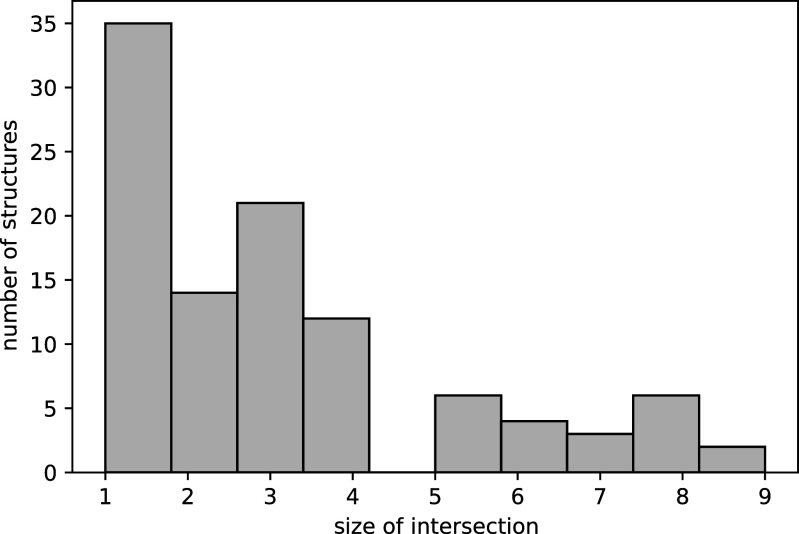
Comparison between 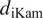 _,_ computed from simulated images, and the Zernike metric, computed from volumes. Here, we repeat simulated experiments 100 times. Then, the size of the intersection of the top ten structures returned by 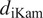 and the Zernike metric is plotted as a histogram.

For every structure, we recover the ground truth as one of the first six lowest-scoring molecules. Moreover, 88 of the 100 tests recovered the ground truth as the lowest-scoring molecule. To evaluate how well the metrics agree on structural similarity, we compute the size of the intersection between the top ten structures returned by our metric and those returned by the Zernike metric. As shown in Figure 6, we find that the metrics agree on two to three structures, and a large number of structures agree only on the ground truth structure. When they occur, disagreements between the metrics are likely due to the presence of near-identical molecules in the database.

### Toward matching experimental datasets by 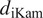


4.4.

While our simulated result shows success in matching a synthetic cryo-EM dataset to PDB structures, many experimental cryo-EM datasets are corrupted by a large number of unmodeled effects that we have not considered. Among the real-data effects are scattering potential’s corruption by a solvent effect,^(^^30^ ^)^ the B-factor,^(^^31^ ^)^ a global scaling ambiguity, imperfect centering, junk particles, non-radial CTF, and imperfect noise model. Our simulation falls short on these counts.

In a first step toward applying 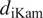 to real experimental datasets, we compare the moments of a stack of images deposited in the Electron Microscopy Public Image ARchive (EMPIAR)^(^^32^ ^)^ to the moments of its preprocessed 3D reconstructions given by the program CryoSPARC^(^^33^ ^)^. We select the dataset EMPIAR-10076,^(^^34^ ^)^ a heterogeneous dataset containing five major structures. The dataset is well characterized, and each image in the dataset has been classified into one of the five major states^(^^34^ ^)^ or “junk” particles, which we discard. We use the classification to generate five separate datasets, allowing us to compute five different moments, one for each of the major states. This test case allows us to examine our metric matching on a real dataset, while bypassing some of the issues associated with comparing datasets and volumes obtained in different experimental conditions.

We downsample the image stack to 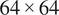 , center using the deposited shift, and mask the images with a circular binary mask of radius 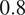 times half the side length of the image. We then estimate the moments for each structure and compare them to moments computed analytically from preprocessed volume reconstructions of the five major structures, as well as two other distinctly shaped ribosomes from the Electron Microscopy Data Bank^(^^35^ ^)^ (EMDB), EMD-8457 and EMD-2660, used as a baseline. Scaling issues between the moment computed from the images and the moment computed from the volume are resolved by examining the diagonal entries of the second moments. Specifically, we find a multiplicative scaling factor that best matches the diagonal of the image-computed second moment and those of the volume-computed second moment under a uniform distribution with respect to the 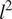 norm.

**Figure 7.**
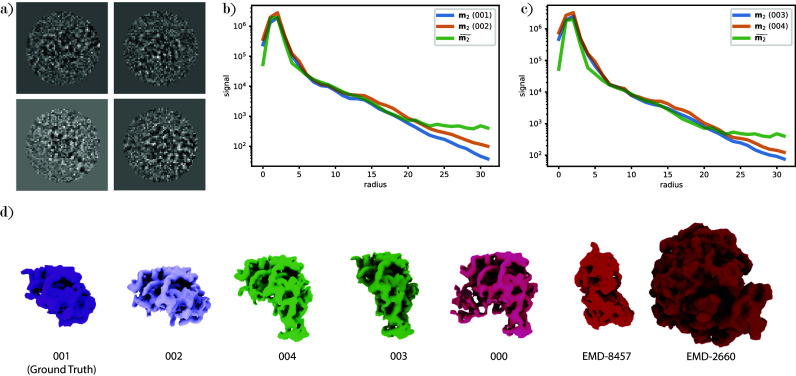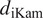 visualization and ranking results for experimental data corresponding to structure 001 (a) Experimental images from EMPIAR-10076 corresponding to structure 001 downsampled to 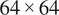 pixels, centered, and with binary mask applied. (b) Comparison between diagonal entries of the second moment computed from the reconstructed volumes 001 and 002 and the moment estimated from experimental images corresponding to structure 001. (c) Comparison between diagonal entries of the second moment computed from the reconstructed volumes 003 and 004 and the moment estimated from experimental images corresponding to structure 001. (d) The five reconstructions (000–004) and two baseline structures (EMD-8457 and EMD-2600) ranked using 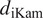 , ordered from left to right.

As shown in Figure 7, it is observed that Kam’s metric recovers the ground truth structure at the lowest distance for the experimental images corresponding to structure 001. We note that the scores for molecules 001 and 002, as well as molecules 003 and 004, are almost identical in value. Also, we find that the analytical moments are closer to each other than to the experimentally determined moments. Finally, the metric reports the baseline structures, which are very different in shape and size, at the largest distances.

**Figure 8.**
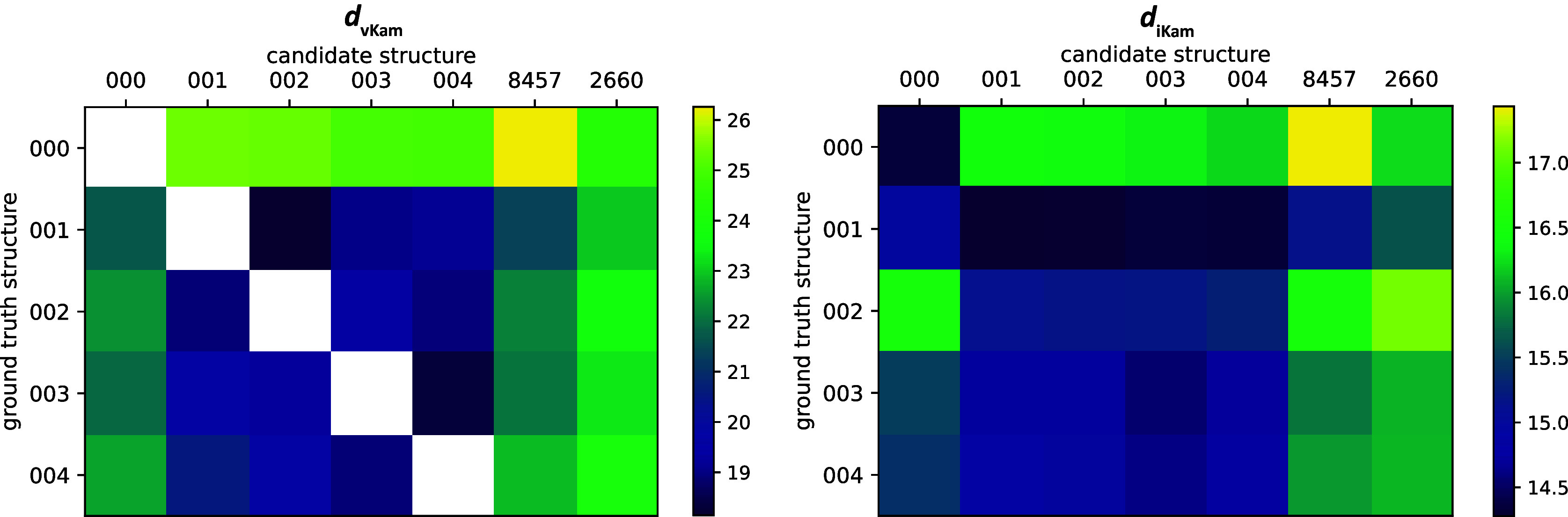
Visualization of 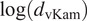 and 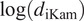 values on the seven candidate structures. Here, EMD-8457 and EMD-2660 are listed as 8457 and 2660 for brevity. Note that there are five ground truth structures but seven candidate structures since EMD-2660 and EMD-8457 are baseline structures for which there are no images in the experimental dataset.

In Figure 8, we plot the distances between the five reconstructions (or in the case of 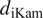 , their experimental images) and the seven candidate structures given by both of our metrics. The exact values for 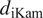 are given in Appendix B.4. There is also scaling ambiguity in 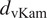 since our reconstructions are preprocessed; hence, we use the same approach as above: We scale each candidate structure’s moment by a multiplicative scaling factor that best matches the candidate structure’s diagonal entries of the second moment with those of the ground truth structure. Analyzing the trends in each row, we observe that the metrics seem to agree on the general ranking of the molecules. While the structures 001, 002 and 003, 004 are very similar, 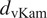 shows that the metric distinguishes between them given accurate moment estimation, whereas 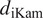 loses some discriminative power. However, when it comes to distinct molecules such as EMD-8457 and EMD-2660, both metrics agree on their rankings.

## 5. Limitations and future work


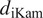
 currently falls short of being directly applicable to experimental datasets. As stated in Section 4.4, there are several unmodeled effects not considered in this work that could lead to unexpected results for real data. The net effect of ignoring these experimental considerations is to bias our moment estimator, which may explain the inability of 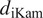 to detect the smaller differences between structures 001 and 002, as well as 003 and 004. Developing an estimator that is robust to outliers (such as junk particles) could help alleviate this.

While we address a few of these parameters, we do so with prior knowledge. For example, the shifts used to center images are a byproduct of the reconstruction process. In future work, we aim to develop methods to correct these effects directly from the raw images. Likewise, here we have controlled for experiment-specific artifacts by using images and structures resolved from the same experiment, whereas in the future we wish to compare across all structures. Furthermore, in the future we seek to compare moments computed from real data directly to the PDB, by appropriately correcting for the discrepancies between PDB and reconstructed structures.

Even with our current mitigations, issues such as the B-factor and inaccuracies in the noise model remain completely unmodeled. Further studies will be required to investigate which of these omissions is important and which can safely be made. Then, our method could be modified to account for the important effects.

## 6. Discussion

We introduced structural similarity metrics for proteins based on moments, inspired by the moment computation in Kam’s method. 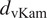 compares known 3D structures according to the difference between the moments of their potentials. We showed that the metric accurately captures similarity according to the rotationally aligned Euclidean metric, an interpretable but expensive-to-compute molecular similarity metric. Therefore, 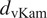 allows for the efficient comparison of large number of known structures. A potential application is to improve the similarity search presently in the PDB, which uses the Zernike metric – a fast but less principled metric that involves learning weights and which our results suggest performs worse than ours.

A second metric, termed 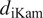 , allows for the computation of a similarity score between an unknown structure present in a large cryo-EM dataset and a solved structure. The computation of this metric does not require a 3D reconstruction process for the image stack and therefore is very efficient. We showed on simulated projection images that our method could discriminate between even very similar proteins with reasonably sized datasets. If it were to work on experimental datasets, 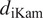 could become a versatile tool for 3D reconstruction. Typical reconstruction algorithms used in practice are only locally optimal and thus require good initialization, which 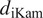 could provide by returning the homologous structures present in the PDB. By extending the database to the entirety of the PDB and including structure predictions, both solved and predicted structures could be quickly compared against.

Beyond its application to experiments, 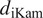 demonstrates that Kam’s method is a feasible strategy for high-resolution reconstruction. Recent works have improved the viability of Kam’s method by using sparsity^(^^7^ ^)^ or neural network^(^^36^ ^)^ priors; likewise, the search over the PDB using Kam’s metric can be interpreted as simply running Kam’s method under a very strong prior, where only a finite number of structures appear with nonzero probability. Our results suggest that, if one could formulate a tractable prior over the manifold of proteins, Kam’s method could yield high-resolution reconstructions.

## Figures and Tables

**Figure B1.**
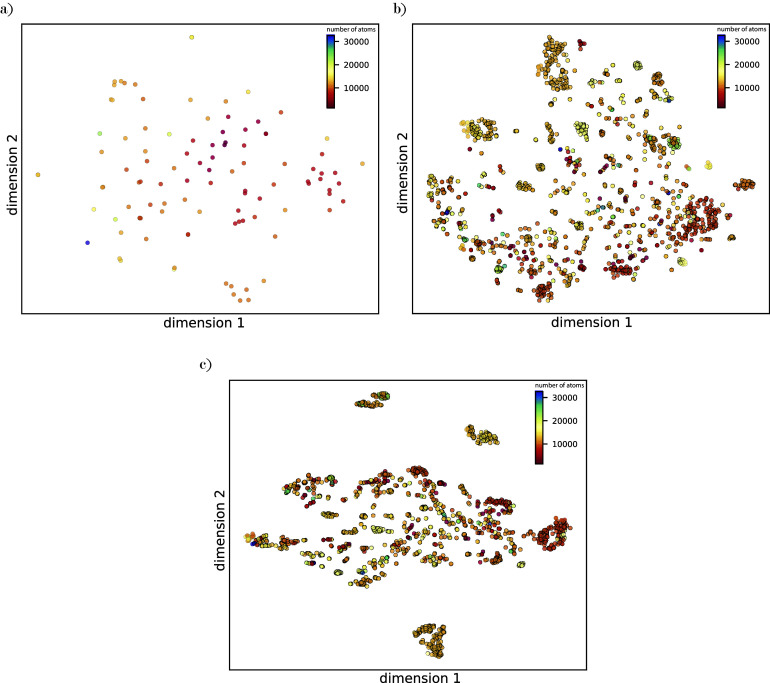
Additional t-SNE plots. (a) t-SNE plot of pairwise Euclidean alignment distances on a subset of size 100. (b) t-SNE plot of pairwise Zernike distances on the entire database. (c) t-SNE plot of pairwise 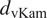 distances on the entire database.

## Data Availability

Replication code can be found at https://github.com/aszhang107/moment-based-metrics/.

## Appendix

### A. Methodology

In this section, we describe the computational details of the method.

#### A.1. Moment derivation

Prior work^(^^8^ ^)^ has shown that the analytical first and second moments of cryo-EM images generated by  and  equal

(12)

where the sum ranges over  such that  ,  is even, and  , and

(13)

where

(14)

is an explicitly calculated constant,

(15)

is a product of Clebsch–Gordan coefficients,^(^^37^ ^)^ and the sum ranges over those indices  that satisfy

(16)

See Sections 2.3.1 and 2.3.2 in,^(^^8^ ^)^ respectively, for the derivations of (12) and (13). In the case of the uniform density on  , we note that  so (12) and (13) simplify to the following:

(17)

(18)

where  is the Legendre polynomial of degree  and  denotes the complex conjugate of a complex number  . The simplification of (12) to (17) is immediate, whereas the simplification of (13) to (18) uses the sum rule for spherical harmonics; see (10) in the study by Kam.^(^^1^ ^)^

#### A.2. Uniform case

This section details the method to compute  . Our algorithm takes as input a PDB identifier (a list of atomic coordinates), on which we center the atomic positions by subtracting the molecule’s center of mass. Then, we use the three-dimensional nonuniform fast Fourier transform (NUFFT)^(^^38^ ^,^^39^ ^)^ to compute the discrete Fourier transform evaluated on a grid in spherical coordinates, that is, to compute

(19)

where  denotes the coordinates of the  atom from the PDB identifier and  is the total number of atoms. The function  is the Fourier transform of the scattering potential of the  atom as reported in the study by Peng et al. and Singer.^(^^40^ ^,^^41^ ^)^ In real space, this corresponds to convolving a Gaussian mixture with a delta function, in other words, adding a Gaussian blob around the atom coordinate. Here,  ,  _,_ and  for  and  and  is the side length of the volume grid in angstroms.

Lastly, we apply the spherical harmonic transform to  defined on the spherical coordinate grid  in (19) using SHTools.^(^^42^ ^,^^43^ ^)^ This gives us coefficients

Let  denote the uniform density on the sphere. In the discrete case, we sample each image as a 2D polar grid at  radial points  and  angular points  , where  is the number of pixels of one side of the projection images. In (12), the first moment  is indexed by  and is thus an  length vector. Note that in (5),  for  , but since  in (13) is  periodic, we have that  _,_ and hence,  . Thus,  for  is redundant and we consider only  for  , which enumerates  . Thus,  is a three-dimensional tensor of size  , since there are  values for  and  values each for  and  , where  are points uniformly spaced between 0 and  and  and  are  uniformly spaced points between 0 and  . Equations (17) and (18) give

(20)

(21)

We then compute the metric given in (10). To better approximate the  norm in the continuous case, we scale the difference of each entry  by  so that the squared norm is scaled by  . More precisely, we define weighted  norms  and  on  and  , as described in (5). Let  and  be two different molecules, and  and  be the first and second moment tensors, respectively, from two different molecules. We define the distance between the moments as in (6).

#### A.3. Least squares for the nonuniform case

This section describes the process for generating and solving the least-squares system for  , the matrix encoding the viewing angle distribution. We use the following convention for the vectorization operator  : If  ,  returns a vector of dimension  obtained by stacking the columns of  , that is,

(22)

The first moment is linear in  as shown in (12), so fitting a viewing distribution to observed moments can be solved through a least-squares problem. We detail this procedure in Algorithm 1. Algorithm 1: Computation of least-squares matrix  for  .

1. initialize  .
2. **for**  **do.**
3. **for**  **do.**
4. **for**  do.
6. **end.**
7. **end.**
8. **end.**
9. **return**  .

Algorithm 2: Computation of least-squares matrix  for  .

1. Initialize  .
2. **for**  **do.**
3. **for**  **do.**
4. **for**  .
5. **for**  .
7. **end.**
8. **end.**
9. **end.**
10. **end.**
11. **return**  .

For the second moment, we rewrite (13) more compactly:

(23)

where

(24)

is a matrix of size  indexed by  and  , and  is a matrix of spherical harmonic coefficients indexed by  . Here, the sum ranges are detailed in (16). Since  is linear in  , we use Algorithm 2 to construct many linear systems  such that

Using the Kronecker product, (23) can be written as

This, too, is linear in  :

(25)

By vertically appending  and copies of  for all values of  in Section 3.1, we obtain the least-squares formulation

where

(26)

To solve this, we perform  decomposition  and then solve the normal equations

that is, we solve  . Since  is a square upper triangular matrix, we solve this using back substitution.

#### A.4. Change of bases for moment comparison

We compute moments from images using the fast method^(^^44^ ^)^ that produces the moments expanded in the Fourier Bessel basis. Thus, a change of bases is required for moment comparison. The Fourier Bessel basis has several nice properties that make it advantageous to use when computing the moment from images; it is orthonormal, frequency-ordered, steerable, provides fast radial convolutions, and has a fast transform.^(^^45^ ^)^ The Fourier Bessel basis functions can be written in polar coordinates  as

(27)

where  is a Bessel function of the first kind of order  , and  is the  -h smallest positive zero of  , and  is a normalization constant.

We create a change of basis matrix  by sampling on a Cartesian grid  with the  grid defined as in Section 3.1, where  are the grid points  in polar coordinates. This yields the moments in real space

Now, we compute the NUFFT to convert the moments into radially sampled polar coordinates in Fourier space as in (19). In practice, we do this for  by taking each row (which is indexed by  ), reshaping it into an image, and applying the transform. We then apply the same process to the columns indexed by  .

#### A.5. Computational complexity

In the following,  is the molecule bandlimit, and see (1);  is the distribution bandlimit, and see (2);  is the number of projection images; and  is the image side length of the  pixel images. We assume that  .

There are three main steps for calculating the least-squares matrix for each structure in our database. We first calculate the least-squares matrices  for  as described in alg:BLSalg. This needs only to be done once and does not need to be recomputed for each molecule. Calculating this matrix takes  time and uses  space. For the calculation of the least-squares matrix itself, we precompute  for  as described in (25). These intermediate steps take  time and use  space for forming the Kronecker product and subsequent matrix multiplication. Finally, the construction of the least-squares matrix  in (26) takes  time for the scalar multiplication of a matrix for each  , and the least squares use  space. As such, the total computational complexity for calculating a least-squares matrix is  time and  space.

The computation of moments from noisy projection images in the Fourier–Bessel basis takes  time and uses  space. To convert this to polar coordinates in Fourier space, we must first evaluate the moments in the Fourier–Bessel basis. This takes  time for each expansion, and we require  such expansions (see app:basis). Hence, in total, this step takes  time and uses  space. Converting into Fourier space using the NUFFT takes  time and uses  space, and we do this  times for a total of  time and space complexity. Storing the final moment uses  space (since the resulting matrix from the NUFFT is block circulant). Overall, computing moments from images and converting them to polar coordinates in Fourier space take  time and use  space.

#### A.6. NDCG score

The NDCG^(^^24^ ^)^ is calculated by taking the discounted cumulative gain (DCG) and normalizing by ideal discounted cumulative gain (IDCG):

(28)

where  is enumerated in the order induced by the predicted scores.

The NDCG puts weight on scores that are agreed to be high by both metrics. However, our metric and the metrics we compare to are dissimilarity scores, so we prefer weight on scores that are considered low by both metrics. To remedy this, we use the reverse of the order enumerated by the predicted scores. For the true scores, we first normalize the scores to the range  and then take the exponential  for each true score  .

### B. Additional Results

#### B.1. Parameter selection

In the experiments, we set the bandlimit parameters to  and  . Note that this value of  is comparable to previous work as described in the study by Sharon et al.^(^^8^ ^)^, whereas the higher value of  allows for a more accurate representation of the molecule in spherical harmonics. Furthermore, the hyperparameter  was set to be 1. As shown in tab:lambda below, varying  does not greatly impact the performance of the metric.

**Table B1.** Effect of the value of the hyperparameter  on the ranking induced by

					
1e-2	**7VV3**	7VUZ	7TRK	7TRP	7VDM
e-1	**7VV3**	7VUZ	7TRK	7TRP	7VDM
1	**7VV3**	7VUZ	7TRK	7TRP	7VDM
e1	**7VV3**	7VUZ	7TRK	7TRP	7VDM
e2	**7VV3**	7VUZ	7TRK	7TRP	7VDM
e3	7Y15	6 K41	7VDM	7VUZ	7E33

*Note*:  *denotes the structure with the*  *th lowest value of*  *In each row, the entry shaded green indicates the ground truth structure.*

#### B.2. Additional t-SNE plots

This appendix includes t-SNE visualizations of the Zernike metric on our database and the alignment metric on a subset of our database of size 100. The alignment metric is restricted to a subset of size 100 since calculating pairwise distances for a 1420 is computationally taxing; see Section 4.2. Visually, the Zernike t-SNE seems to have fewer distinct clusters than the t-SNE plot generated using  and also groups molecules with different numbers of atoms together. It seems possible that Zernike metric is less discriminative, although this may also be an artifact of t-SNE’s dimensionality reduction.

#### B.3. Robustness to number of images

Here, we examine the robustness of our metric to inaccuracies of moment estimation. Specifically, we vary the number of noisy synthetic projection images that the metric has access to and record the highest-ranking structures.

**Table B2.** *Effect of the number of projection images used for moment estimation on the ranking induced by*

Number of images						Relative error (%)	Relative error (%)
500	7VDM	7VUZ	7Y15	7E33	**7VV3**	1.49	8.23
1000	7VUZ	7VDM	**7VV3**	7TRK	7EJ8	0.76	6.22
2500	7VUZ	7VDM	**7VV3**	7TRK	7TRP	0.43	4.02
5000	**7VV3**	7TRK	7VUZ	7TRP	7VDM	0.27	2.95
10000	**7VV3**	7VUZ	7TRK	7TRP	7VDM	0.25	1.89
25000	**7VV3**	7VUZ	7TRK	7VDM	7TRP	0.19	1.37
50000	**7VV3**	7VUZ	7TRK	7VDM	7TRP	0.14	1.15

*Note*:  *denotes the structure with the*  *th lowest value of*  . *In each row, the entry shaded green indicates the ground truth structure. The relative error in each moment is between the moment estimated from noisy projection images and the moment calculated from their clean counterparts.*

#### B.4. Additional experimental results

Table B3 reports the metric’s rankings using experimental images corresponding to the five structures resolved from EMPIAR-10076.

**Table B3.** Performance of  on structures 001, 002, 003, 004, and 005 of EMPIAR-10076

Number of images							
2018	**000** (14.56)	004 (16.22)	EMD-2660 (16.23)	003 (16.32)	002 (16.42)	001 (16.45)	EMD-8457 (17.43)
12650	**001** (14.28)	002 (14.29)	004 (14.34)	003 (14.35)	000 (14.95)	EMD-8457 (15.15)	EMD-2660 (15.64)
26104	001 (15.13)	**002** (15.16)	004 (15.18)	003 (15.29)	000 (16.50)	EMD-8457 (16.51)	EMD-2660 (17.14)
26138	**003** (14.56)	004 (14.73)	001 (14.74)	002 (14.74)	000 (15.50)	EMD-8457 (15.83)	EMD-2660 (16.08)
36561	003 (14.62)	**004** (14.80)	001 (14.84)	002 (14.88)	000 (15.40)	EMD-8457 (16.00)	EMD-2660 (16.09)

*Note*:  *denotes the structure with the*  *th lowest value of*  . *The value of*  *is reported next to each structure in parenthesis. In each row, the entry shaded green indicates the ground truth structure.*
